# Radial peripapillary capillary changes and retinal nerve fiber layer alterations in diabetic foot ulcers with mild-to-moderate nonproliferative diabetic retinopathy: an OCTA study

**DOI:** 10.55730/1300-0144.6147

**Published:** 2025-11-17

**Authors:** Eyüpcan ŞENSOY, H. Erhan GÜVEN, Mehmet ÇITIRIK

**Affiliations:** 1 Department of Ophthalmology, Ankara Etlik City Hospital, University of Health Sciences, Ankara, Turkiye; 2 Department of General Surgery, Ankara Etlik City Hospital, University of Health Sciences, Ankara, Turkiye

**Keywords:** Diabetic foot ulcers, diabetic retinopathy, radial peripapillary capillary, retinal nerve fiber layer, microvascular changes

## Abstract

**Background/aim:**

This study aimed to evaluate the impact of diabetic foot ulcers (DFUs) on retinal microvascular changes, specifically the radial peripapillary capillary (RPC) vessel density and retinal nerve fiber layer (RNFL) thickness in patients with mild-to-moderate nonproliferative diabetic retinopathy (NPDR).

**Materials and methods:**

Ninety participants were enrolled and divided into three groups: 30 patients with DFUs and mild-to-moderate NPDR, 30 patients with NPDR but without DFUs, and 30 healthy controls. RPC density and RNFL thickness were evaluated using optical coherence tomography angiography (OCTA), a noninvasive imaging method. Comparisons between groups were made with statistical adjustments, including optic disc area.

**Results:**

Patients with DFUs exhibited significantly lower RPC vascular density across multiple quadrants than those without DFUs and healthy controls (p < 0.001). Furthermore, RNFL thickness was significantly increased in the DFU group, particularly in the inferior-hemi (p < 0.001) and temporal-inferior (p < 0.001) quadrants. The optic disc area was significantly larger in the DFU group (p = 0.017), which may have influenced the RNFL measurements.

**Conclusion:**

The study underscores notable alterations in RPC density and RNFL thickness among patients with DFUs and mild-to-moderate NPDR. These alterations may reflect systemic microvascular impairment, potentially exacerbated by systemic inflammation, or changes suggestive of reactive gliosis, though further validation is needed. These findings underscore the need for early ophthalmological evaluation and a comprehensive approach to managing both peripheral and ocular diabetic complications. Future studies incorporating systemic inflammatory biomarkers and functional visual assessments are needed to explore the mechanisms underlying these retinal changes.

## 1. Introduction

Diabetes mellitus (DM) is a chronic metabolic condition that affects a large and growing global population and is associated with various complications, including diabetic foot ulcers (DFUs) and diabetic retinopathy (DR). As the prevalence of DM continues to rise worldwide, these complications remain major contributors to morbidity, disability, and reduced quality of life in individuals with diabetes [1,2]. Despite advances in glycemic control and management strategies that have improved overall outcomes, the coexistence of DFUs and DR presents a complex clinical challenge that warrants further investigation.

DFUs are a common and serious complication of diabetes, primarily resulting from nerve damage, poor peripheral circulation, and a weakened immune response [3,4]. These ulcers are often chronic, with prolonged inflammation, impaired wound healing, and an increased risk of infection, potentially leading to amputation. Similarly, DR is the leading cause of vision loss in diabetic individuals, caused by chronic hyperglycemia that leads to retinal microvascular damage, including capillary nonperfusion, increased vascular permeability, and progressive neuronal injury [5,6]. Both DFUs and DR involve microvascular dysfunction, suggesting that systemic vascular changes may link these two complications.

Although previous studies have noted a bidirectional association between DFUs and DR [7,8], the specific mechanisms underlying this association remain unclear. Systemic inflammation and endothelial dysfunction, common features of DFUs, may contribute to retinal changes, potentially through increased microvascular damage and neuroinflammatory responses [9,10]. This remains a hypothesis and should be interpreted as a possible mechanism rather than established evidence. This study aims to investigate how these factors mediate retinal alterations in patients with DFUs and nonproliferative DR (NPDR). Emerging evidence suggests that systemic inflammation and endothelial dysfunction may contribute to both DFU and DR severity [9,10]. Yet, the retinal alterations associated with DFUs, particularly in the earlier stages of DR (such as mild-to-moderate NPDR), remain understudied.

Optical coherence tomography angiography (OCTA) is a high-resolution, noninvasive imaging technique that allows for precise visualization of the retinal microvascular network and associated neural structures [11,12]. While OCTA is commonly used to analyze the superficial and deep capillary plexuses (SCP/DCP) in DR, the radial peripapillary capillary (RPC) network has received relatively limited attention. This network, which supplies both the retinal nerve fiber layer (RNFL) and the optic nerve head (ONH), is crucial for neurovascular coupling and retinal health [11,13]. Notably, changes in RPC may serve as early indicators of neurovascular dysfunction, making them an important target for the study of diabetic complications.

While previous research has shown alterations in both RPC density and RNFL thickness in DR [12,14], the impact of DFUs on these parameters has not been extensively explored. The pathophysiology of DFUs involves systemic inflammatory processes that can potentially exacerbate retinal microvascular injury. Furthermore, the effects of DFUs on retinal neural structures, particularly the RNFL, remain poorly understood. This study seeks to fill this gap by examining the relationship between DFUs and retinal alterations in RPC vessel density and RNFL thickness, specifically in patients with mild-to-moderate NPDR.

We hypothesized that patients with DFUs would exhibit greater microvascular impairment, reflected by lower RPC vascular density and increased RNFL thickness. Increased RNFL thickness might reflect reactive gliosis or other neuroinflammatory processes; however, further validation is needed. By focusing on the RPC network, this study seeks to provide new insights into how DFUs may influence neurovascular alterations and contribute to retinal deterioration in diabetic patients.

## 2. Methods

This cross-sectional, prospective study was conducted at Ankara Etlik City Hospital, in accordance with the ethical principles outlined in the Declaration of Helsinki. Ethical approval was obtained from the Institutional Ethics Committee (approval no: 2023-258), and written informed consent was collected from all participants before enrollment.

A total of 90 participants were recruited and divided into three groups. Group 1 consisted of 30 patients diagnosed with advanced DFUs, classified as Wagner stage 3 or 4, along with mild-to-moderate NPDR. Group 2 included 30 patients with mild-to-moderate NPDR but without DFUs. Group 3 comprised 30 healthy individuals, age- and sex-matched, with no history of diabetes or ocular disease.

Participants with the following conditions were excluded from the study: type 1 diabetes, proliferative DR (PDR), diabetic macular edema (DME), recent ocular surgery or laser treatment (within the past six months), intraocular injections (within the last six months), refractive errors greater than ±2.0 diopters, poor OCTA image quality (signal strength index <7), and ocular conditions such as amblyopia, hypertensive retinopathy, retinal vein occlusion, or a history of ocular trauma. Additionally, participants with PDR were excluded to avoid confounding effects of advanced retinal neovascularization, which could significantly influence OCTA measurements and complicate the assessment of microvascular changes related to DFUs in earlier stages of retinopathy.

To classify the presence of mild-to-moderate NPDR, diabetic patients were assessed and categorized using the International Clinical Diabetic Retinopathy Severity Scale. Inclusion criteria required participants to be 18 years of age or older with a confirmed diagnosis of type 2 DM and mild-to-moderate NPDR or to be healthy individuals with no history of diabetes.

**Figure 1:**
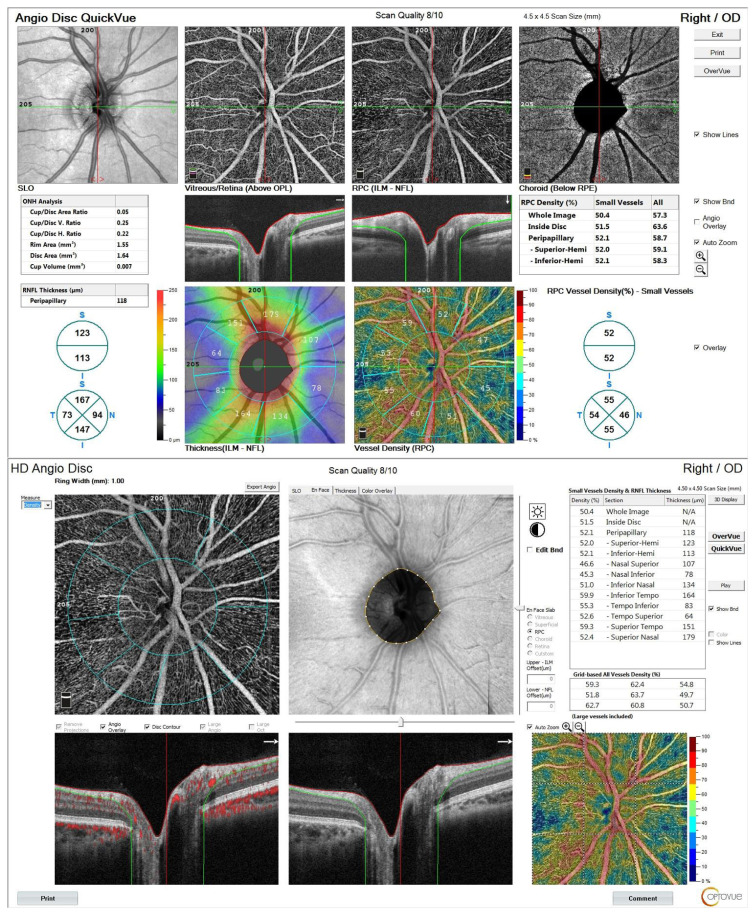
Representative topographic mapping of RNFL and RPC quadrants in a healthy eye.

All participants underwent a comprehensive ophthalmological examination, including best-corrected visual acuity (BCVA), intraocular pressure measurement, and refractive status assessment. OCTA was conducted using the AngioVue system (RTVue XR Avanti; Optovue, Fremont, CA, USA), employing a 4.5 × 4.5-mm scanning area centered on the optic nerve head (ONH). The following OCTA parameters were assessed: RPC vascular density, measured across the inside of the disc; the whole image; the peripapillary region; and specific quadrants: nasal-superior, inferior-nasal, temporal-inferior, and temporal-superior. RNFL thickness was measured in multiple quadrants: peripapillary, inferior-hemi, nasal-superior, inferior-nasal, temporal-inferior, and temporal-superior. ONH parameters were also evaluated, including optic disc area, cup-to-disc ratio, and rim area. A schematic diagram of the measurement quadrants (Figure 1) has been added to clarify the anatomical regions analyzed.

Images with artifacts or segmentation errors were excluded after review by an experienced ophthalmologist to ensure data quality. Automated segmentation and quantitative analyses were performed using software embedded in the OCTA device.

The study focused on RPC vascular density, as this layer directly supplies the RNFL and optic nerve head, both of which are critical to diabetic retinal neurovascular pathology. While the SCP and DCP are commonly evaluated in DR studies, we specifically focused on the RPC to investigate its potential role in the retinal changes associated with DFUs.

### 2.1. Statistical analysis

Statistical evaluations were performed using SPSS software (version 23.0; IBM Corp., Armonk, NY, USA). The Shapiro–Wilk test was utilized to assess the normality of continuous variable distributions. For data following a normal distribution, comparisons between groups were made using one-way analysis of variance (ANOVA), followed by Bonferroni-adjusted post hoc analyses. When the data did not meet normality assumptions, the Kruskal–Wallis test was used, with subsequent pairwise comparisons conducted using the Mann–Whitney U test and the Bonferroni correction. Categorical variables were analyzed using the Pearson chi-square test.

A post hoc power analysis based on the observed differences in RPC density yielded a power of 0.82 at α = 0.05, supporting the adequacy of the sample size (n = 30 per group).

To account for the potential confounding effect of optic disc area, partial correlation analyses were performed with optic disc area included as a covariate. Formal analysis of covariance (ANCOVA) or multivariate regression modeling was not applied in this dataset but is planned for future studies to enable more robust adjustment. Statistical significance was defined as p < 0.05.

## 3. Results

The clinical and demographic characteristics of the study groups are summarized in Table 1. The average age of participants in the DFU + NPDR group (58.9 years) was slightly younger than that of those in the NPDR-only (60.7 years) and healthy control groups (61.7 years). The DFU group had a higher proportion of males (76.7%) compared with the other groups. Additionally, the DFU + NPDR group had a significantly larger optic disc area (2.02 ± 0.35 mm^2^) compared with both the NPDR-only (1.84 ± 0.44 mm^2^) and healthy control groups (1.85 ± 0.30 mm^2^) (Table 1).

**Figure 2:**
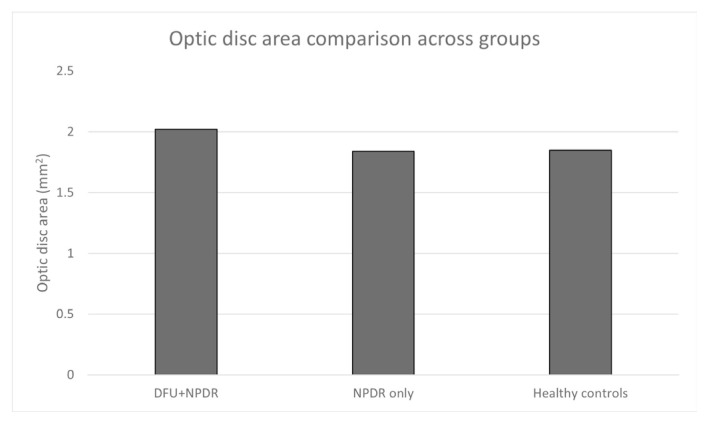
Optic disc area comparison across groups.

The optic disc area was significantly larger in the DFU + NPDR group compared with the NPDR-only and healthy control groups (p = 0.017), as shown in Figure 2.

**Figure 3:**
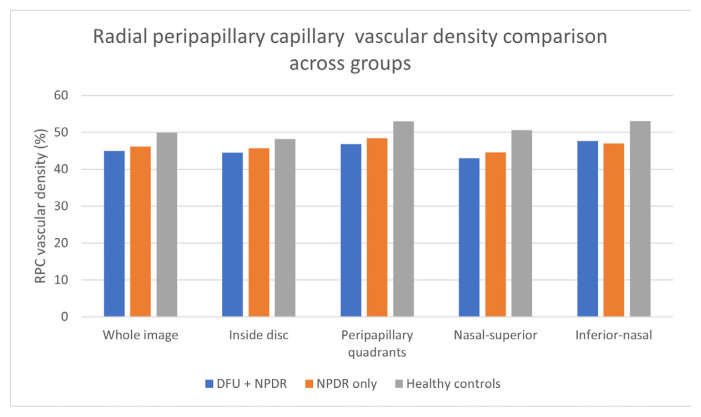
RPC vascular density comparison across five quadrants and groups. across five quadrants and groups. disc area comparison across groups.

Significant reductions in RPC vascular density were observed in the DFU + NPDR group across multiple retinal regions. The RPC density in the DFU group was notably lower than in both the NPDR-only group and healthy controls across all quadrants, as illustrated in Figure 3 and Table 2. The DFU group had a mean RPC vascular density of 45.0% ± 2.9 in the whole image, compared with 46.2% ± 2.7 in the NPDR-only group and 49.9% ± 3.0 in healthy controls (p < 0.001).

**Figure 4:**
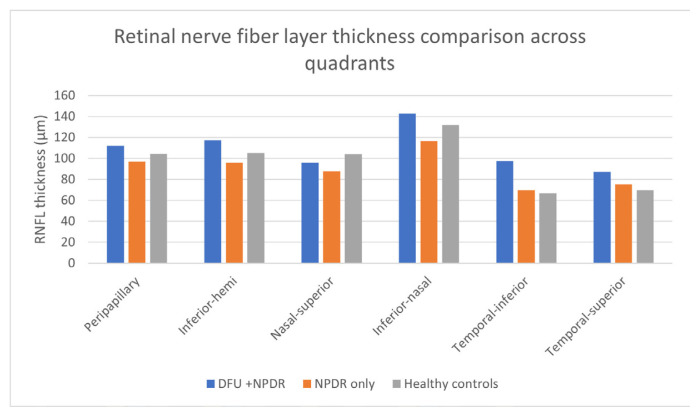
RNFL thickness comparison across six quadrants.

The DFU + NPDR group exhibited significantly increased RNFL thickness compared with both the NPDR-only group and healthy controls. This difference was most pronounced in the peripapillary, inferior-hemi, temporal-inferior, and inferior-nasal quadrants (Figure 4). For example, the average peripapillary RNFL thickness in the DFU group was 112.0 ± 20.1 μm, which was significantly greater than that observed in the NPDR-only (96.8 ± 15.2 μm) and healthy control groups (104.5 ± 10.4 μm) (p = 0.003).

## 4. Discussion

DFUs and DR are two major complications of diabetes. However, the relationship between these conditions, particularly regarding retinal microvascular changes and neurodegenerative alterations, has not been fully explored. The primary objective of this study was to investigate the association between DFUs and changes in RPC vessel density and RNFL thickness in individuals with mild-to-moderate NPDR. Our results demonstrate that patients with both DFUs and NPDR show significant differences in RPC density and RNFL thickness compared with diabetic individuals without DFUs and healthy controls. These findings suggest underlying microvascular compromise and potentially reactive gliotic changes.

We observed that patients with DFUs had significantly reduced RPC vascular density across multiple retinal regions, including within the disc, the whole image, and the peripapillary areas, compared with both NPDR-only patients and healthy controls. This finding aligns with previous research indicating that individuals with diabetes experience retinal microvascular alterations, such as capillary loss and decreased vascular density, linked to systemic vascular dysfunction [11,13]. The reduction in RPC vascular density in the DFU group may reflect the systemic inflammatory state induced by chronic wounds and impaired wound healing, both of which are known to contribute to endothelial dysfunction and microvascular damage in diabetic patients [9,10]. Although statistically significant, the magnitude of these differences was relatively small, and their clinical relevance remains uncertain. Longitudinal studies are warranted to determine whether such subtle microvascular alterations translate into meaningful functional outcomes. Nevertheless, the RPC network, which supplies both the RNFL and optic nerve head, may still serve as a sensitive marker of retinal microvascular injury in individuals with diabetic complications.

In the inferior nasal quadrant, RPC vascular density did not significantly differ between the DFU + NPDR and NPDR-only groups, suggesting that the presence of DFUs did not exert an additional local microvascular effect in this region. However, both diabetic groups showed markedly lower values than healthy controls (p < 0.001), indicating generalized diabetic microvascular impairment rather than DFU-specific alteration. These findings highlight that DFU-related microvascular compromise may not affect all peripapillary sectors uniformly.

One of the most striking findings of this study was the significantly increased RNFL thickness in the DFU group, particularly in the peripapillary, inferior hemi, and temporal inferior quadrants. This increase in RNFL thickness may seem counterintuitive, as diabetic neurodegeneration is typically associated with RNFL thinning [14]. However, as suggested by previous studies, increased RNFL thickness in diabetes might indicate early neuroinflammatory changes, such as reactive gliosis, an inflammatory response involving Müller cells, astrocytes, and microglia. However, this interpretation remains speculative and requires further histopathological or biomarker-based confirmation.

In patients with DFUs, the systemic inflammation underlying chronic wounds may exacerbate retinal neuroinflammation, contributing to the observed changes in RNFL thickness. Our results align with the growing literature linking DFUs to neuroinflammatory processes in the retina, suggesting that these patients may be at risk of developing both peripheral and ocular neurovascular damage. Furthermore, increased RNFL thickness in DFU patients may contribute to altered visual function, as observed in some diabetic individuals. However, visual field testing and electrophysiological assessments, which were not part of this study, are necessary to confirm this hypothesis. Alternative explanations, such as measurement artifacts, anatomical variations (e.g., larger optic disc area), or localized edema, could also contribute to the apparent RNFL thickening.

We also observed a significantly larger optic disc area in the DFU group, which may have confounded the interpretation of RNFL thickness. A larger optic disc area can lead to thicker RNFL measurements due to anatomical variation, as larger discs tend to have a more extensive RNFL. Although we included optic disc area as a covariate to minimize its influence on RNFL measurements, we acknowledge that advanced statistical methods, such as ANCOVA or multivariate regression, would provide a more rigorous adjustment. Future studies will employ these approaches to confirm the observed associations and reduce potential bias caused by anatomical variation [15].

This study has several limitations. Its cross-sectional design prevents establishing causality between DFUs and retinal changes, and the clinical relevance of the statistically significant differences in RPC density (e.g., 45.0% vs. 46.2%) is uncertain. Future longitudinal studies with larger sample sizes and functional assessments are needed to determine if these alterations progress with disease severity and to evaluate their clinical impact.

Moreover, this study did not include an evaluation of systemic inflammatory indicators such as C-reactive protein (CRP), interleukin-6 (IL-6), and tumor necrosis factor-alpha (TNF-α). These biomarkers could provide deeper insights into the biological mechanisms contributing to the observed retinal alterations. Another important limitation is that axial length was not measured. Since axial length can influence RNFL thickness, this omission should be taken into account when interpreting the results. A sex imbalance was observed in the DFU group (76.7% male), which may introduce bias due to known sex-related differences in RNFL thickness. Although statistical adjustment for sex was not performed in this study, future studies should consider this factor when analyzing retinal changes in diabetic populations. Furthermore, excluding individuals with PDR and DME limits the generalizability of the findings. Including patients with PDR and DME in future studies would provide a more comprehensive understanding of the influence of DFUs on retinal structure across all stages of DR. Although this study focused on structural changes, the absence of functional visual assessments, such as visual field testing or electrophysiological studies, limits the clinical relevance of our findings. Integrating these tests into future studies would provide a direct correlation between structural retinal changes and visual function, offering a more complete understanding of how DFUs and NPDR affect vision.

Given the limitations of the cross-sectional design, future studies should use longitudinal cohorts to clarify better the time-dependent relationships among DFUs, NPDR, and retinal changes. Incorporating systemic inflammatory markers and functional visual assessments will be essential to elucidate the mechanisms underlying these retinal alterations and their clinical implications. Longitudinal studies should also examine the role of inflammation in retinal microvascular and neurodegenerative changes in diabetic patients with DFUs. Additionally, studies that include patients with proliferative DR and diabetic macular edema will help broaden our understanding of how DFUs influence retinal changes across different stages of DR.

This study offers new insights into retinal alterations in individuals with mild-to-moderate NPDR and DFUs. The results show that patients with DFUs exhibit significantly lower RPC density and increased RNFL thickness compared with both diabetic individuals without DFUs and healthy controls. These alterations may reflect systemic microvascular impairment and possibly reactive gliosis; however, further studies are required to substantiate this hypothesis.

Due to the limited and homogeneous distribution of DFU stages (only Wagner stages 3 and 4 were included) and unequal subgroup sizes, correlation analyses between DFU stage and retinal changes were not performed. This represents a limitation of the study, and we plan to explore this in future, larger-scale studies with more evenly distributed DFU stages.

The larger optic disc area observed in DFU patients highlights the importance of considering anatomical variations when interpreting RNFL measurements. It is well established that larger optic discs are often associated with thicker RNFL measurements, reflecting the greater number of nerve fibers distributed over a wider area. The significantly larger optic disc area observed in the DFU group (p = 0.017) may therefore partially contribute to the higher RNFL thickness measurements. This anatomical factor should be considered when interpreting the results, particularly in cross-sectional analyses.

These findings may suggest the need for early ophthalmological evaluation in patients with DFUs, as retinal microvascular changes may indicate systemic microvascular dysfunction. However, further studies are required to validate these associations. OCTA could serve as a valuable noninvasive tool for assessing retinal health in this high-risk population. Nevertheless, the routine clinical implementation of OCTA for early screening should be approached cautiously and supported by evidence from larger, longitudinal, multicenter studies. Future studies incorporating systemic inflammatory biomarkers and functional assessments are crucial for further exploring the mechanisms linking peripheral and ocular diabetic complications and improving patient management.

## Figures and Tables

**Table 1:** Demographic and clinical characteristics of study groups.

Characteristic	DFU + NPDR (Group 1)	NPDR Only (Group 2)	Healthy controls (Group 3)	p-value*
**Age (years)**	58.86 ± 8.3	60.74 ± 10.5	61.73 ± 6.97	0.20^K^
**Sex (female)**	7 (23.3%)	11 (36.7%)	12 (40%)	0.35^P^
**Sex (male)**	23 (76.7%)	19 (63.3%)	18 (60%)	
**Duration of diabetes (years)**	14.43 ± 8.5	13.93 ± 8.26	-	0.76^M^
**Wagner stage (Group 1)**	Stage 3: 7 (23.3%)	-	-	
	Stage 4: 23 (76.7%)	-	-	
**Optic disc area (mm** **^2^** **)**	2.02 ± 0.35	1.84 ± 0.44	1.85 ± 0.30	0.02^K^
**Duration of NPDR (years)**	5.3 ± 3.6	5.5 ± 4.2	-	0.84^M^

Data are presented as mean ± standard deviation (range) or number (%). NPDR, nonproliferative diabetic retinopathy; DFU, diabetic foot ulcer.

* K: Kruskal–Wallis H test; Mann–Whitney U test; P: Pearson chi-square test

**Table 2:** RPC vascular density comparison among groups.

Quadrant	DFU + NPDR (Group 1)	NPDR Only (Group 2)	Healthy controls (Group 3)	p-value*
**Whole image**	45.0 ± 2.9	46.2 ± 2.7	49.9 ± 3.0	<0.001^K^
**Inside disc**	44.5 ± 4.8	45.7 ± 5.4	48.2 ± 4.1	0.027^A^
**Peripapillary**	46.8 ± 3.5	48.4 ± 4.3	53.0 ± 3.6	<0.001^K^
**Nasal-superior**	43.0 ± 4.8	44.6 ± 5.9	50.6 ± 4.8	<0.001^K^
**Inferior-nasal**	47.7 ± 5.5	47.0 ± 6.1	53.1 ± 4.7	<0.001^K^

Data are presented as mean ± standard deviation.

* A: One-way ANOVA test; K: Kruskal–Wallis H test

## Data Availability

The datasets generated during and/or analyzed during the current study are available from the corresponding author on reasonable request.
